# Large-Scale Screens of miRNA-mRNA Interactions Unveiled That the 3′UTR of a Gene Is Targeted by Multiple miRNAs

**DOI:** 10.1371/journal.pone.0068204

**Published:** 2013-07-09

**Authors:** Peng Zhou, Weiyi Xu, Xueling Peng, Zhenhua Luo, Qinghe Xing, Xulin Chen, Chengqian Hou, Weihong Liang, Jianwen Zhou, Xiaoyan Wu, Zhou Songyang, Songshan Jiang

**Affiliations:** 1 State Key Laboratory of Biocontrol, School of Life Sciences, Sun Yat-sen University, Guangzhou, China; 2 Key Laboratory of Gene Engineering of the Ministry of Education, School of Life Sciences, Sun Yat-sen University, Guangzhou, China; 3 Institutes of Biomedical Sciences, Fudan University, Shanghai, China; 4 Gene Science & Health company, Shenzhen, China; 5 College of Life Sciences, Henan Normal University, Xinxiang, China; IPMC, CNRS UMR 7275 UNS, France

## Abstract

Animal microRNA (miRNA) target prediction is still a challenge, although many prediction programs have been exploited. MiRNAs exert their function through partially binding the messenger RNAs (mRNAs; likely at 3′ untranslated regions [3′UTRs]), which makes it possible to detect the miRNA-mRNA interactions *in vitro* by co-transfection of miRNA and a luciferase reporter gene containing the target mRNA fragment into mammalian cells under a dual-luciferase assay system. Here, we constructed a human miRNA expression library and used a dual-luciferase assay system to perform large-scale screens of interactions between miRNAs and the 3′UTRs of seven genes, which included more than 3,000 interactions with triplicate experiments for each interaction. The screening results showed that the 3′UTR of one gene can be targeted by multiple miRNAs. Among the prediction algorithms, a Bayesian phylogenetic miRNA target identification algorithm and a support vector machine (SVM) presented a relatively better performance (27% for EIMMo and 24.7% for miRDB) against the average precision (17.3%) of the nine prediction programs used here. Additionally, we noticed that a relatively high conservation level was shown at the miRNA 3′ end targeted regions, as well as the 5′ end (seed region) binding sites.

## Introduction

MicroRNAs (miRNAs) are a class of small single-strand non-coding RNAs with a common length of about 22 nucleotides (nt) [1]. MiRNAs usually play a role in posttranscriptional regulation of coding genes by partially complementing with targeting mRNAs [1], [2]. The miRNA target site has been considered to be the 3′ untranslated region (3′UTR) of a mRNA, however, recent studies have shown that miRNAs may also bind the coding regions or the 5′ untranslated regions (5′UTRs) [3], [4]. In animals, when a miRNA binds to its target mRNA, it usually inhibits gene translation and sometimes degrades the mRNA [5], [6]. MiRNAs widely exist in plants and animals and the number of hairpin precursor miRNAs was updated to 21,264 in miRBase 19, which was made public in August 2012 [7]. The functions of miRNAs are involved in most biological processes (e.g., development [8], [9]) and in disease pathogenesis (e.g., cancer [10], [11]). Discovery of the miRNA target genes is urgently needed for functional and mechanical study of these small RNAs.

MiRNA target prediction is often used to determine the candidate target genes for experimental verification. Unlike plant miRNAs, which are always perfectly complementary to their target genes [12], animal miRNAs are often partially complementary to the target mRNAs, which makes it more difficult to predict miRNA-mRNA interactions. Many prediction programs have been developed since miRNA was discovered [1]. The first generation of miRNA target prediction programs were designed based on a hypothesis (e.g., seed complementary, binding free energy and site conservation), such as TargetScan [13], [14], DIANA_microT [15] and miRanda [16], [17]. Since each program contains different features, the overlap between each prediction result has been quite low [18]. To get a better prediction result, several bioinformatic methods were introduced into the second generation of prediction programs, such as the hidden Markov model (HMM) [19], support vector machine (SVM) classifier [20], [21] and the Bayesian phylogenetic model [22]. In addition, the number of predicted target genes has been increased.

It is important to experimentally evaluate the performance of the prediction programs and to choose the correct prediction programs. A commonly used method for validation of predicted interactions is a dual-luciferase assay through co-transfection of the luciferase reporter gene containing the target 3′UTR and synthetic miRNA mimics or a miRNA expression vector, which has been used to confirm predicted interactions in small scale studies [1], [23], [24], [25]. However, there is no report on using this approach in large-scale or genome-wide studies. Recently, several new approaches have been developed to identify the miRNAs and targets on large-scale, including proteomic methods, co-IP based experiments and miRNA transfection methods [26]. In the proteomic approach, the capability of mass spectrometry to identify and quantify proteins from complex mixtures depends on the level of accuracy and sensitivity [27]. For co-IP based methods, an antibody that recognizes a protein (usually an Agonaute protein) is used to profile the target mRNA [28]. In addition, miRNA transfection that combined with other approaches including transcriptomic or proteomic analysis has been widely used to identify miRNA targets [26].

In the present study, we presented large-scale screens for 3′UTRs in seven genes, which included more than 3,000 interactions with triplicate experiments individually, using a co-transfection and dual-luciferase assay system approach. The gene 3′UTRs cloned into the multiple cloning regions were located at 3′ end to the *Renilla* luciferase gene. If a miRNA targeted its binding site in the 3′UTR of the gene, the activity of *Renilla* luciferase would be decreased. We tested 1,018 interactions predicted by computer programs and 2,433 interactions screened by a genome-wide miRNA expression library. We demonstrated that the 3′UTR of a gene can be targeted by multiple miRNAs and many of them cannot be predicted using popular prediction programs.

## Materials and Methods

### Vector Construction

To express miRNA, a pLL3.7 vector was first modified by the insertion of an overlap extension PCR (OE-PCR) product containing multiple cloning sites, the miRNA transcriptional stop sequence 5′-TTTTT-3′ and the puromycin resistant gene driven by the SV40 early promoter, downstream of the human U6 promoter. The SV40 early promoter was PCR amplified from pcDNA3.1 using the forward primer (primer 1, 5′- CTCGAGAACCCGGGATCCTTTTTATTTAACGCGAATTAATTCTGTGGAA-3′) with XhoI, SmaI and BamHI restriction sites (underlined) followed by a transcriptional stop sequence of the U6 promoter, 5′-TTTTT-3′, at the 5′ terminus. The reverse primer (primer 2, 5′- TACTCGGTCATGCTAGCCGGGAGCTTTTTGCAAAAGCCTAG-3′) with an 11 nt overlap sequence of puromycin PCR products was followed by a NheI restriction site (underlined) linked at the 5′ terminus. The puromycin resistant gene was amplified from pCDH-CMV-MCS-EF1-Puro_CD510B-1 using a forward primer (primer 3, 5′- GCAAAAAGCTCCCGGCTAGCATGACCGAGTACAAGCCCACGGTGC-3′) that contained a 14 nt overlap sequence with the SV40 early promoter PCR product and a NheI restriction site (underlined). The reverse primer (primer 4, 5′-TTTTTGTCGACGAATTCTCAGGCACCGGGCTTGCGGGTCATGC-3′) included SalI restriction sites. Then an overlap extension PCR (OE-PCR) was carried out with a combination of the above two PCR fragments and amplification with primers 1 and 4. The final PCR product was digested with SalI and inserted into the pLL3.7 plasmid that was digested by HpaI and XhoI. This modified pLL3.7 was named pLE. To construct the human miRNA expression library, a genomic fragment containing the human miRNA precursor and flanking sequences was amplified for each miRNA and cloned into the pLE vector using XhoI and BamHI double digestion.

To construct luciferase reporter vectors, the psiCHECK-2 (Promega) vector was modified by deletion of the BamHI site between the firefly luciferase gene and β-lactamase (Amp^r^) coding region, the multiple cloning region was altered to contain XhoI and BamHI sites. The full-length 3′UTRs of seven human genes *MXI1*, *TP53*, *PTEN*, *CYP3A4*, *FSCN1*, *POT1* and *TRF2* or the 3′UTR fragments containing the putative miR-101-3p or miR-10b-5p binding sequences of human *EZH2* or *HoxD10* were amplified using the primers listed in **Table S1** and were cloned downstream of the *Renilla* luciferase in the modified psiCHECK-2 vector using XhoI/SalI and BamHI/BglII double digestion. The mutant luciferase reporter constructs carrying mutations in the sequence of the complementary miRNA seed region sites were generated by site-directed mutagenesis using overlap extension PCR, as described previously [29].

The miRNA sensor was made by tandemly linking 2 or 4 copies of complementary sequences of the mature miRNA together and they were cloned downstream of the *Renilla* luciferase (Rluc) gene in a modified psiCHECK-2 vector, as described by Ebert *et al.* [30]. The synthesized oligos for sensor cloning are listed in **Table S2**.

### Cell Culture and Dual-luciferase Reporter Assay

The human HEK-293T (293T) cell line, purchased from the American Type Culture Collection (ATCC, MD), was cultured in DMEM/High Glucose medium (Thermo) supplemented with 10% fetal bovine serum (FBS, Thermo) and 100 U/ml penicillin/streptomycin (Thermo) in 5% CO_2_ at 37°C.

The dual-luciferase reporter assay was modified, as previously described [29]. Briefly, 2.5×10^4^ 293T cells in 100 µL growth medium were plated in 96-well plates. The next day, the cells were transfected with a 150 ng miRNA expression vector or empty vector and a 50 ng luciferase reporter vector containing full-length 3′UTRs of *MXI1*, *TP53*, *PTEN*, *CYP3A4*, *FSCN1*, *POT1* or *TRF2,* or the 3′UTR fragment of *EZH2* or *HoxD10* fused to the *Renilla* luciferase reporter gene using FuGene HD (Roche). The co-transfection of the miRNA empty vector and the same luciferase reporter vector was used as a control and transfections were performed for each plate. The cells were harvested 48 h after transfection and assayed using the Dual-Luciferase Reporter Assay Kit (Promega) according to the manufacturer’s instructions. Each transfection was repeated in triplicate.

The ratio of *Renilla* luciferase activity to firefly luciferase activity in each well was normalized to the average ratio of the control wells in each plate in which the ratio was designed as 1. The data for the same 3′UTR reporter collected from different plates were integrated by combination of the normalized ratios. All experimental data are presented as the mean±S.D. from three independent transfection experiments. Significant differences from the control value analyzed with Student’s t-test are indicated by *P*<0.05.

### RNA Isolation and Quantitative Real-time PCR

The total RNA of 293T cells that were transfected with a miRNA empty vector, pre-miR-24 or pre-miR-27a was isolated by Trizol (Invitrogen). One microgram of RNA was used to synthesize the first strand complementary DNA (cDNA) with Rever-Tra-Ace-α- (TOYOBO) and random primers. Quantitative real-time PCR (qPCR) was performed in a StepOnePlus Real-Time PCR System (Applied Biosystems) with SYBR Premix ExTaq II (TaKaRa) to detect miR-24-3p, miR-27a-3p and internal control U6 RNA. The primers were purchased from GenePharma. Each sample was tested in triplicate and the expression fold change was determined using the ΔΔC_T_ method [31].

### Immunoblotting Analysis

293T cells were transfected with a miRNA empty vector or various miRNA expression vectors. After 72 h, cells were harvested and lysed by RIPA lysis buffer (Bioteke) with 1 mM proteinase inhibitor PMSF. Total proteins were separated by 10% SDS-PAGE gels, and subsequently transferred onto Polyvinylidene fluoride (PVDF) membranes (Millipore). The membrane was blocked with 5% nonfat dry milk in TBS-T buffer for 1 hour and incubated overnight at 4°C with antibodies against MXI1 (1∶500, Santa Cruz) or β-actin (1∶5000, Abcam). Followed by washing in TBS-T, the membrane was incubated with horseradish peroxidase (HRP)-conjugated secondary antibody for 1 hour at room temperature, respectively. Finally, the signal was detected with SuperSignal West Pico Chemiluminescent Substrate (Thermo Scientific).

### Data Analysis

Triplicate experiments were performed for each interaction. Significant differences between each interaction and the control group were determined with a Student *t*-test. *P*<0.05 was considered as statistically significant. The inhibition effect was shown by counting the percentage of each luciferase-activity ratio taken in the control groups. A modified SSMD method was used for statistical analysis of the genomic miRNA screening results [32]. Interactions that produced a value equal to or less than -2 were considered as strong downregulation effects, except as specifically indicated.

### MiRNA Prediction

Nine programs were chosen to predict the miRNAs that targeted the 3′UTRs of seven genes (*MXI1*, *TP53*, *PTEN*, *CYP3A4*, *FSCN1*, *POT1* or *TRF2*). For TargetScan, TargetScanS, miRanda, miRDB and PicTar, predictions were determined using their web servers with default parameters. For microT_v3.0, the threshold 7.3 was used to predict miRNAs; for NBmiRTar, the threshold score was 0.9; for PITA, “use filler upstream sequence” was chosen, and the minimum seed size was set at 8 nt, single G:U pair and single mismatch in the seed region was allowed, “3 upstream/15 downstream” in the “flank settings” was set and the cutoff value was −10; for EIMMo, the cutoff score for medium confidence of predicted miRNAs was a p score of 0.5. After prediction, 18–75% of the total predicted miRNAs for each gene were randomly chosen for experimental validation of the putative miRNA-mRNA interactions by a dual-luciferase activity assay.

### Analysis of the Prediction Precision of the Programs

Prediction precision was the percentage of the validated miRNA-mRNA pairs in the putative miRNA-mRNA pairs. For each program, the precision was calculated with each gene first and then the combined validated interactions and putative interactions were used to calculate the total prediction precision. The same method was used to calculate the co-prediction precision.

### Analysis of miRNA Recognition Elements

The sequences of the 3′UTRs of target genes, including predicted miRNA recognition elements (MREs), 24 nt of 3′ end flanking sequence and 10 nt of 5′ end flanking sequence, were extracted. The complementary sequence of the miRNA seed region was located in the center. All of the tested interactions that could be predicted by at least one program are listed in **Table S3**. They were divided into the positive group (n = 101) and negative group (n = 875). 203 sequences with 42 nt in length randomly chosen from the human genomic DNA were referred to as the random group. To compare the conservation level of the MREs in each group, Vertebrate Cons (phastCons44ways) [33] in the UCSC web service was used. The significant difference was tested with a Wilcoxon test. The miRNA target site distribution was obtained using the UCSC Genome Browser.

## Results

### Construction of a miRNA Expression Library

**Figure 1:**
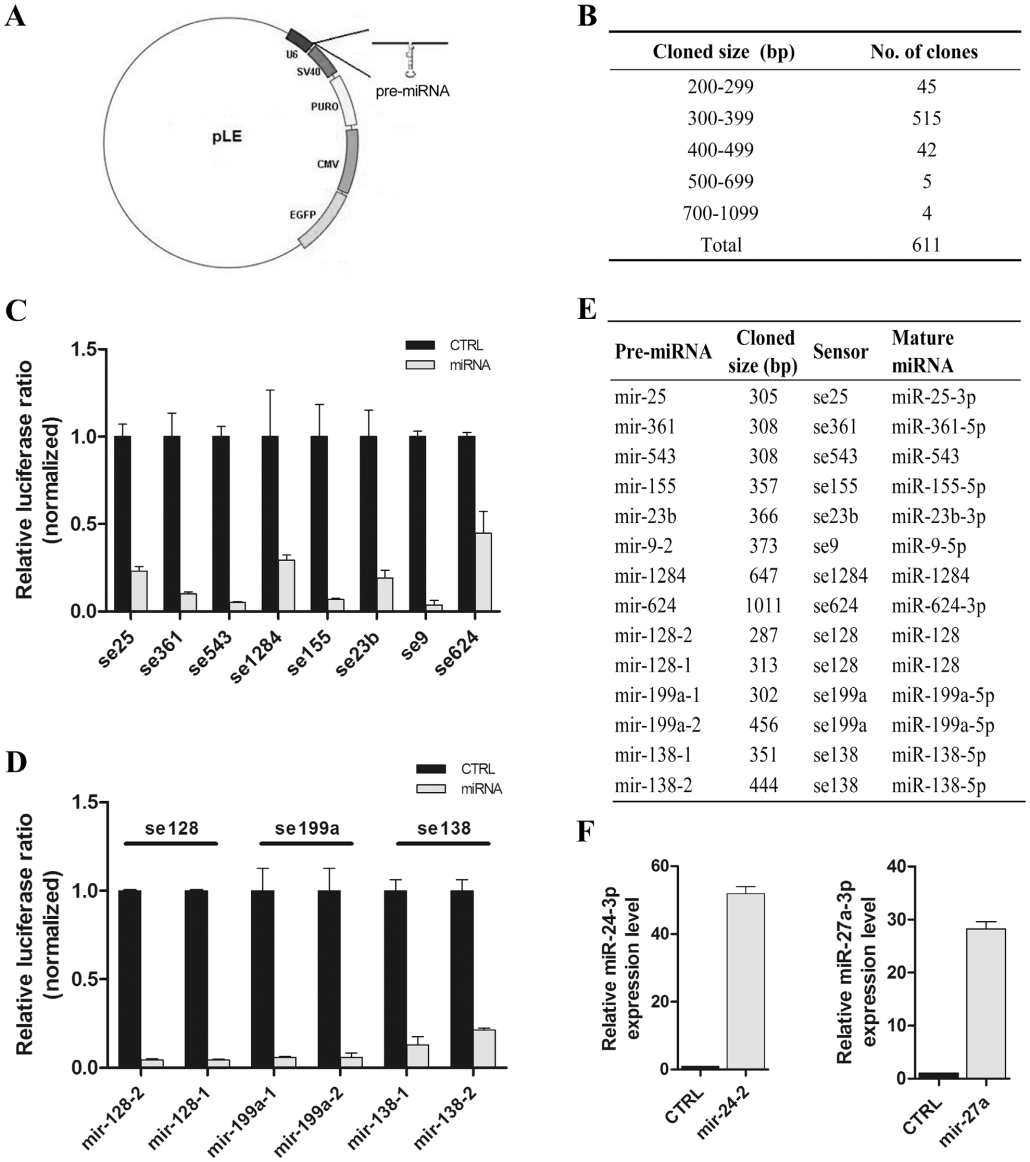
The human miRNA expression library. (A) The map of the miRNA expression vector contains the puromycin resistance gene and EGFP reporter gene. The miRNA precursor and flanking sequences were cloned into the downstream of the U6 promoter (see Materials and Methods). (B) The size distribution of the inserts containing each miRNA precursor and flanking genomic sequences in the constructed miRNA expression library. (C) and (D) The pre-miRNA expression vector and cognate miRNA sensor were co-transfected in 293T cells to test whether the construct could express the miRNA triple repeats for each experiment (p<0.05). The results of one sensor corresponding to one pre-miRNAgenomic locus are shown in (C) and the results of one sensor corresponding to two pre-miRNA genomic locus are shown in (D). The co-transfection of the empty vector of miRNA and the same sensor was used as a control. (E) The detailed information of each sensor and tested miRNA that were demonstrated in (C) and (D). (F). Relative levels of miR-24-3p and miR-27a-3p miRNAs measured by qPCR for transient transfection of each pre-miRNA expression vector in 293T cells.

To express miRNAs, the pLL3.7 vector was modified as described in the materials and methods section (**Figure 1A**). For efficient expression of mature miRNAs, the length of any flanking sequences at both sides of the miRNA stem-loop structure should be larger than 40 bp [34]. For this reason, all of our PCR products designed to amplify the miRNA precursors contained at least 40 bp of flanking sequences at both sides. According to the miRBase database (http://www.mirbase.org/), we constructed a miRNA expression library consisting of more than 600 human miRNA precursors (i.e., pre-miRNAs), including most of the early found and abundant miRNAs. The distribution of the length of PCR products to amplify these pre-miRNAs is listed in **Figure 1B**. To measure whether our miRNA constructs could efficiently produce mature miRNAs, 15 miRNA sensors were constructed according to the reported method [30] with the synthesized oligos listed in **Table S2**. A miRNA sensor contains a tandem 2 or 4 copies of the complementary sequence of the mature miRNA inserted at the 3′UTR region of the Renilla luciferase (Rluc) gene in the psiCHECK-2 vector (Promega). The results showed that the relative luciferase ratios of the co-transfection of the miRNA expression vector and cognate miRNA sensor vector were obvious low compared with that of the control (**Figure 1C, 1D and 1E**; data not shown for miR-27a-3p, -23a-3p, -342-3p and -24-3p). The miRNA expression vectors, with the length of the cloned pre-miRNA fragments ranging from 287–456 bp, could produce miRNA with high efficiency. Even the constructs with a cloned size of 647 bp for miR-1284 or 1011 bp for miR-624-3p could produce miRNA at a reasonable level (**Figure 1C and 1E**). For those miRNAs with more than one locus in the genome, 3 sensors for miR-128, -138-5p and -199a-5p, each of which has two loci, were used to measure the miRNA expression levels with cloning both genomic loci, respectively. Both the genomic DNA of each miRNA could produce the miRNA quite efficiently (**Figure 1D and 1E**). The quantitative RT-PCR (qRT-PCR) results for miR-24-3p and miR-27a-3p also showed that the expression vector could generate mature miRNA efficiently (**Figure 1F**). Collectively, our results demonstrated that the miRNA expression library we constructed could efficiently be processed into mature miRNAs.

### Measurement of Interactions between miRNAs and Target 3′UTRs

**Figure 2:**
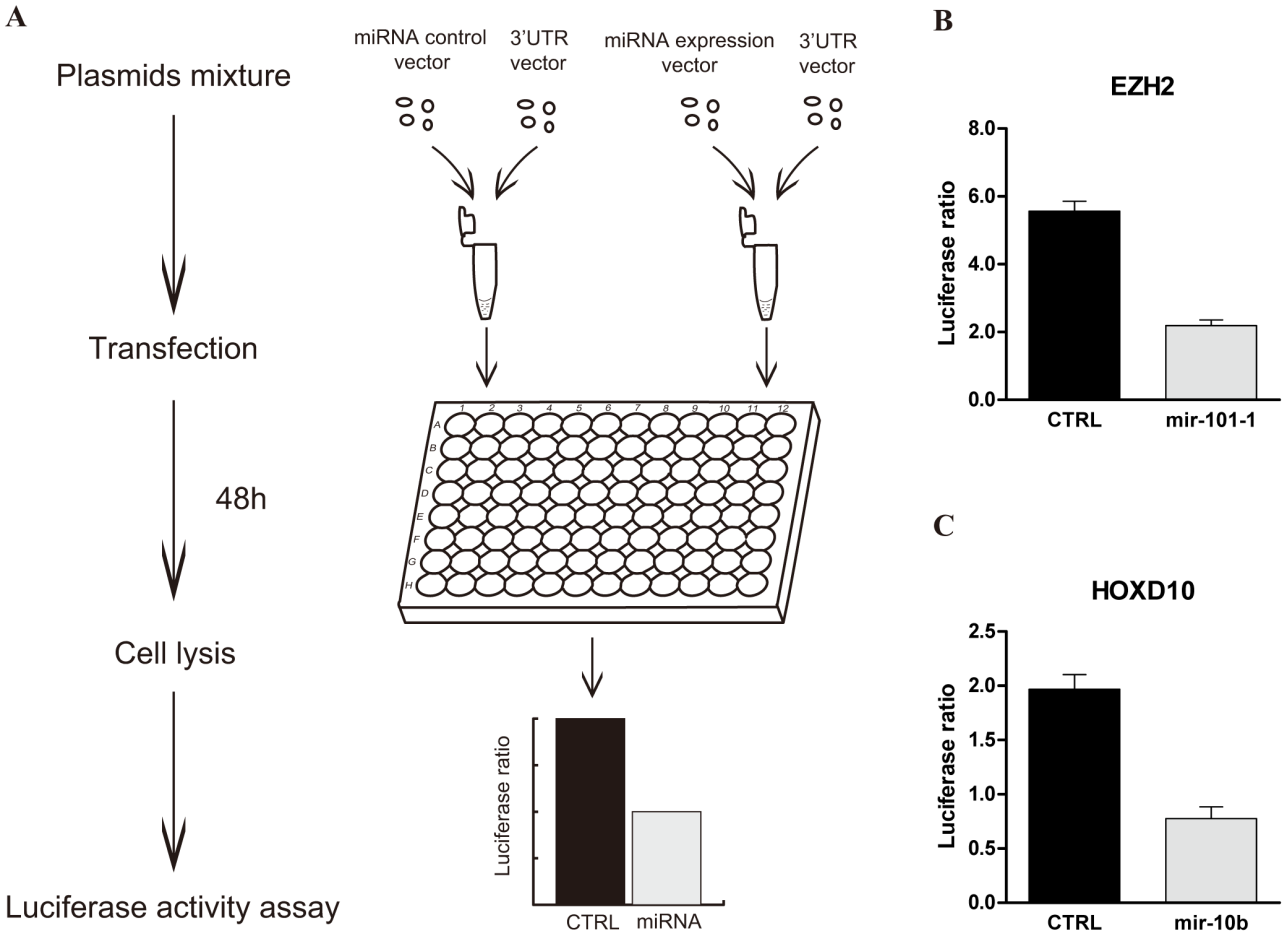
The strategy for validation of the interactions between miRNAs and target gene 3′UTRs. (A) The experiment procedures. (B) and (C) Co-transfection of pre-miRNA (0.15 µg) and the luciferase reporter containing the target gene 3′UTR (0.05 µg) into 293T cells to test the reported interactions of miR-101-3p and *EZH2* 3′UTR (B) or miR-10b-5p and *HoxD10* 3′UTR (C), with co-transfection of the empty vector of pre-miRNA and the luciferase reporter containing the same 3′UTR as the control. The data were normalized to the ratio of *Renilla* and firefly luciferase activities measured at 48 h post transfection. Values represent the mean ± S.D. from three independent transfection experiments.

The target site of miRNA is often located at the 3′UTR [3]. In order to establish a reliable large-scale method to test the interactions between miRNAs and target genes, we first constructed *Renilla* luciferase reporter vectors containing the 3′UTRs to examine two reported interactions (**Figure 2A**). Co-transfections with the miRNA expression vector plus the 3′UTR report vector into 293T cells were performed to detect the interactions. It has been reported that the human enhancer of zeste homolog 2 gene (*EZH2*) is targeted by miR-101-3p in SKBr3 breast epithelial cells, DU145 prostate carcinoma cells and benign immortalized H16N2 breast epithelial cells [35] and homeobox D10 (*HoxD10*) is targeted by miR-10b-5p in SUM149 primary breast carcinoma cells [36]. Our recent study also showed that *HoxD10* is a functional target gene of miR-10b-5p in human microvascular endothelial cells (HMEC-1) involved in heparin inhibition of angiogenesis [29]. In the present study, we showed that the miR-101-3p produced by the constructed vector could inhibit the *EZH2* 3′UTR reporter gene and that miR-10b-5p could inhibit the *HoxD10* 3′UTR reporter gene (**Figure 2B and 2C**).

### Large-scale Screens of Predicted Interactions Revealed Multiple miRNAs Targeting the 3′UTR of Each Gene

To assess the efficiency of miRNA target prediction programs, we performed systematic experiments to validate miRNA-mRNA interactions using our human miRNA expression library and the miRNA-mRNA interaction detecting system. Nine miRNA prediction programs were chosen to perform predictions, including TargetScan [37], TargetScanS [13], [14], PITA [38], DIANA-microT_v3.0 (microT_v3.0) [15], PicTar [19], [39], miRanda [16], [17], miRDB [20], [21], NBmiRTar [40] and EIMMo [22]. These are often used to stand for rule based algorithms and machine learned algorithms (**Table S4**).

**Figure 3:**
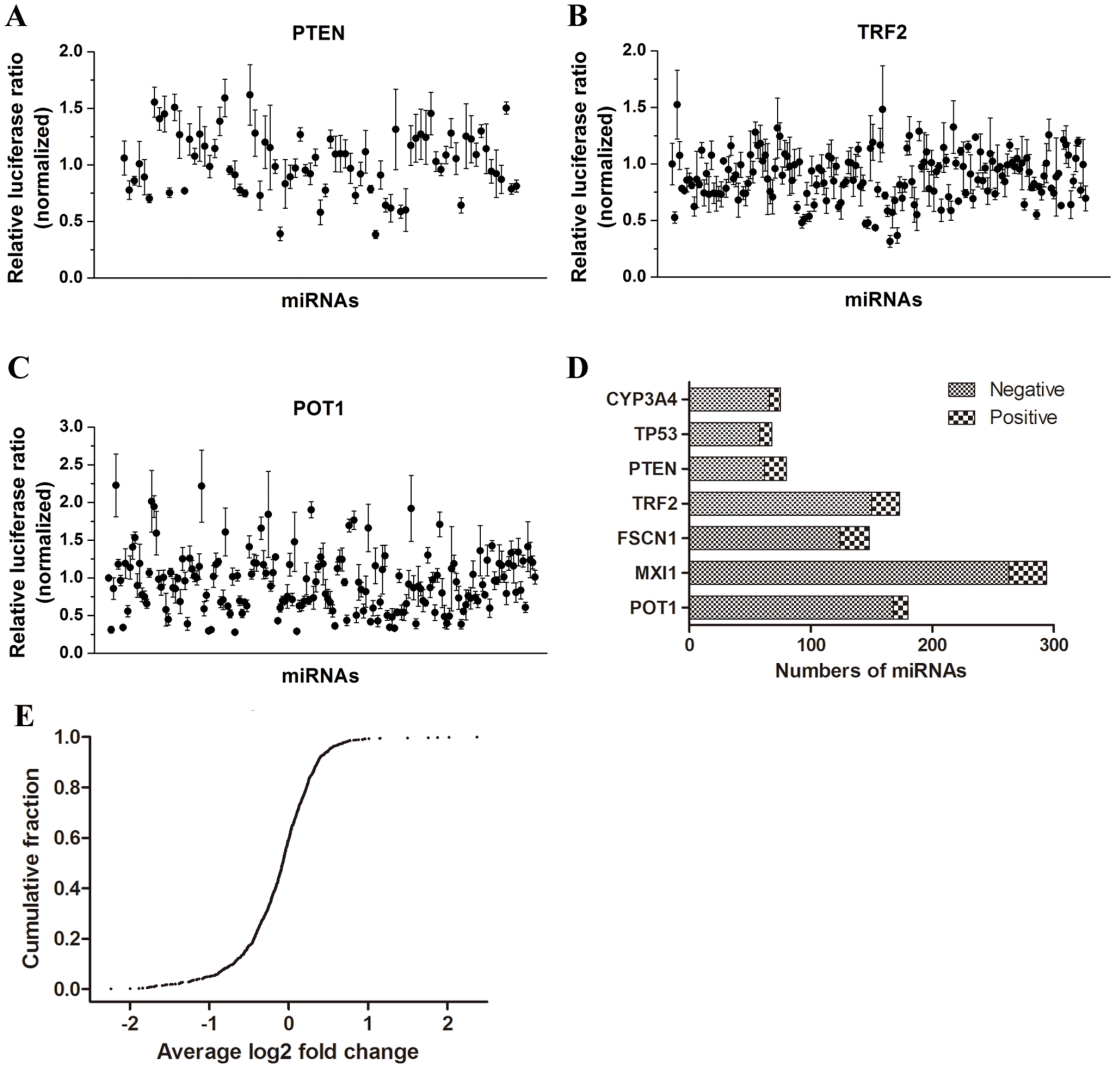
Validation of the predicted interactions for seven genes. (A–C) Co-transfection of 0.15 µg of the pre-miRNA expression vector and 0.05 µg of the luciferase reporter vector containing the full length 3′UTR of *PTEN* (A), *TRF2* (B) and *POT1* (C) into 293T cells to valid the predicted interactions, with co-transfection of the empty vector of the pre-miRNA and the luciferase reporter containing the same 3′UTR as control. The data were normalized to the ratio of *Renilla* and firefly luciferase activities measured at 48 h post transfection. Values represent the mean ± S.D. from triplicate transfection experiments. (D) Summary of the results for each gene based on the tested interactions. Inhibitive miRNAs with *p*<0.05 were chosen as positive. (E) The cumulative curve showed the distribution of fold changes for all the predicted interactions. Fold changes were calculated according to the negative controls in each experiment.

Seven genes, including *MXI1*, *TP53*, *PTEN*, *CYP3A4*, *FSCN1*, *POT1* and *TRF2,* were chosen for miRNA prediction with the above mentioned programs. We chose these genes in order to exclude the effect that genes of similar function share similar miRNA-target profiling. The prediction parameters were described in the methods section and the numbers of predicted miRNAs for each gene were summarized in **Table 1**. The predicted miRNAs from the nine software programs for each gene were combined together to obtain a final list of predicted miRNAs (**Table S3**). These prediction results showed that there was a significant difference for miRNAs and miRNA numbers predicted by the different software programs (**Tables 1** **and S3**). Subsequently, 18–75% of predicted interactions for these 7 genes were randomly chosen for large-scale experimental validation using a dual luciferase assay with a co-transfected miRNA expression vector and cognate 3′UTR reporter vector. Totally, 1,018 predicted interactions were examined. The positive interactions were defined as the data that had statistically significant difference compared to the control experiments that replaced the miRNA expression vector with a miRNA empty vector. The other tested interaction data were classified as negative. The luciferase results indicated that each gene was regulated by multiple miRNAs (**Figure 3**; data not shown), with 3′UTRs of *CYP3A4*, *TP53*, *PTEN*, *TRF2*, *FSCN1*, *MXI1* and *POT1* bound by 9 miRNAs, 10 miRNAs, 18 miRNAs, 23 miRNAs, 24 miRNAs, 31 miRNAs and 12 miRNAs, respectively (**Table 1**). The total number of positive interactions for the 1,018 predicted interactions of the seven genes was 127 (**Figure 3E**).

**Table 1:** Summary of prediction results.

	TargetScan	TargetScanS	PITA	microT_v3.0	PicTar	miRanda	miRDB	NBmiRTar	EIMMo
*MXI1*									
Number of miRNAs predicted	79	117	29	27	14	190	26	126	47
Number of miRNAs tested	71	104	15	26	11	165	25	77	43
Number of miRNAs found positive	11	14	0	3	3	22	7	5	8
Precision (%)	15.5	13.5	0.0	11.5	27.3	13.3	28.0	6.5	18.6
*TP53*									
Number of miRNAs predicted	53	68	82	10	0	8	10	124	9
Number of miRNAs tested	21	31	19	8	0	5	9	34	7
Number of miRNAs found positive	4	4	2	1	0	1	2	6	1
Precision (%)	19.0	12.9	10.5	12.5	0.0	20.0	22.2	17.6	14.3
*PTEN*									
Number of miRNAs predicted	115	177	40	71	14	243	59	181	92
Number of miRNAs tested	26	37	6	20	4	48	15	30	23
Number of miRNAs found positive	5	12	0	7	0	14	8	4	8
Precision (%)	19.2	32.4	0.0	35.0	0.0	29.8	53.3	13.3	34.8
*CYP3A4*									
Number of miRNAs predicted	43	74	43	0	0	0	18	74	3
Number of miRNAs tested	32	43	7	0	0	0	10	10	2
Number of miRNAs found positive	4	4	0	0	0	0	1	3	0
Precision (%)	12.5	9.3	0.0	0.0	0.0	0.0	10.0	30.0	0.0
*POT1*									
Number of miRNAs predicted	45	82	23	6	0	25	19	67	0
Number of miRNAs tested	41	67	12	6	0	21	18	40	0
Number of miRNAs found positive	5	8	0	0	0	1	1	2	0
Precision (%)	12.2	11.9	0.0	0.0	0.0	4.8	5.6	5.0	0.0
*TRF2*									
Number of miRNAs predicted	50	41	65	0	1	79	8	113	8
Number of miRNAs tested	43	34	24	0	1	67	8	67	7
Number of miRNAs found positive	7	8	1	0	0	11	0	8	2
Precision (%)	16.3	23.5	4.2	0.0	0.0	16.4	0.0	11.9	28.6
*FSCN1*									
Number of miRNAs predicted	47	38	27	3	0	8	8	133	8
Number of miRNAs tested	38	31	10	3	0	7	4	73	7
Number of miRNAs found positive	8	9	1	2	0	1	3	8	5
Precision (%)	21.1	29.0	10.0	66.7	0.0	14.3	75.0	11.0	71.4
Summary									
Total number of miRNAs predicted	432	597	309	117	29	553	148	818	167
Total number of miRNAs tested	272	347	93	63	16	313	89	331	89
Total number of miRNAs found positive	44	59	4	13	3	50	22	36	24
Precision (%)	16.2	17.0	4.3	20.6	18.8	16.0	24.7	10.9	27.0

**Figure 4:**
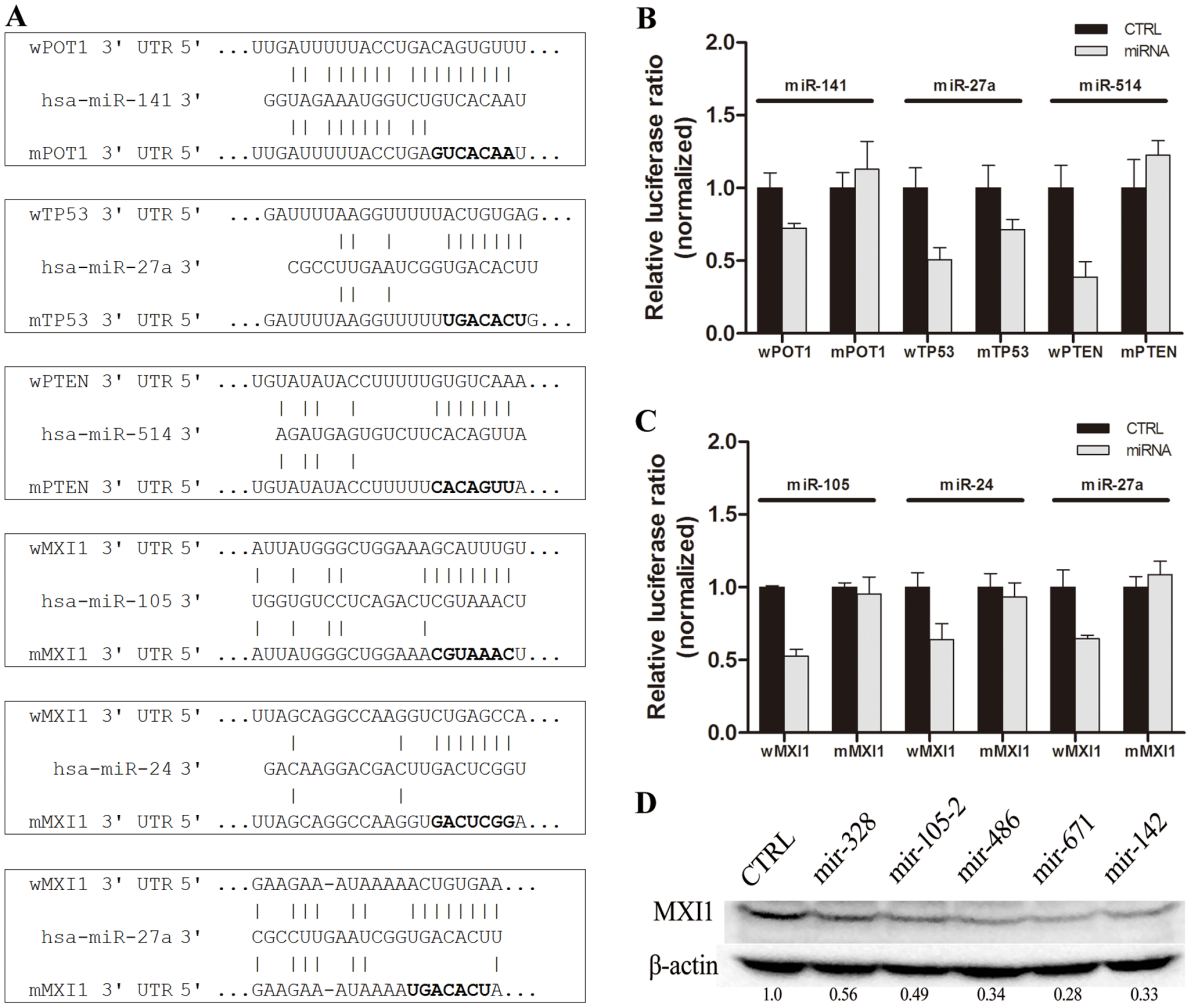
Confirmation of the validated interactions with site mutation and immunoblotting. (A–C) Positive interactions were chosen for mutation analysis of miRNA target sites. The predicted matched sequences between miRNAs and target sites, as well as the seed region chosen for mutant constructs are shown in (A) and the dual-luciferase assay was used to examine the co-transfection of the pre-miRNA and the luciferase reporter containing the cognate target site or mutated cognate target site in *POT1*, *TP53* and *PTEN* (B) or in *MXI1* (C), in which there was co-transfection of the empty pre-miRNA vector and the same luciferase reporter as a control. (D) Immunoblotting was used to examine the endogeous protein level of target gene *MXI1* in 293T cells when the positive miRNAs were transfected into the cells and cultured for 48 h post-transfection.

To examine whether the positive interactions was reliable, some of these interactions were chosen for further confirmation using mutation and immunoblotting analyses. 12 interactions for POT1, TP53, PTEN and MXI1, with 3 interactions for each gene, were randomly chosen for mutation analysis of miRNA target sites. When the complementary sequences of the miRNA seed region in the target sites were mutated, the miRNAs did not interact with the 3′UTRs or the inhibition effect was attenuated (**Figure 4A, B and C**, data not shown). For the further confirmation, we choose half positive interactions of MXI gene using immunoblotting analysis. When the miRNA vectors expressing miRNAs, which had positive interactions with the MXI1 gene 3′UTR, were transiently transfected into the 293T cells, the endogenous MXI1 protein level decreased significantly (**Figure 4D**). These data, as well as many unpublished target site mutation results and immunoblotting results performed in our laboratory, might suggested that the identified positive interactions were reliable.

Our analysis showed that EIMMo had the highest prediction precision (27%), and the second was miRDB (24.7%). The lowest precision was observed from PITA (4.3%). The precisions of other prediction tools were 16.2% for TargetScan, 17% for TargetScanS, 20.6% for DIANA-microT_v3.0, 18.8% for PicTar, 16% for miRanda and 10.9% for NBmiRTar (**Table 1**).

### Genome-wide Screens of *MXI1, POT1, TRF2* and *FSCN1* Revealed Unpredicted miRNAs Targeting the 3′UTR of Each Gene

**Figure 5:**
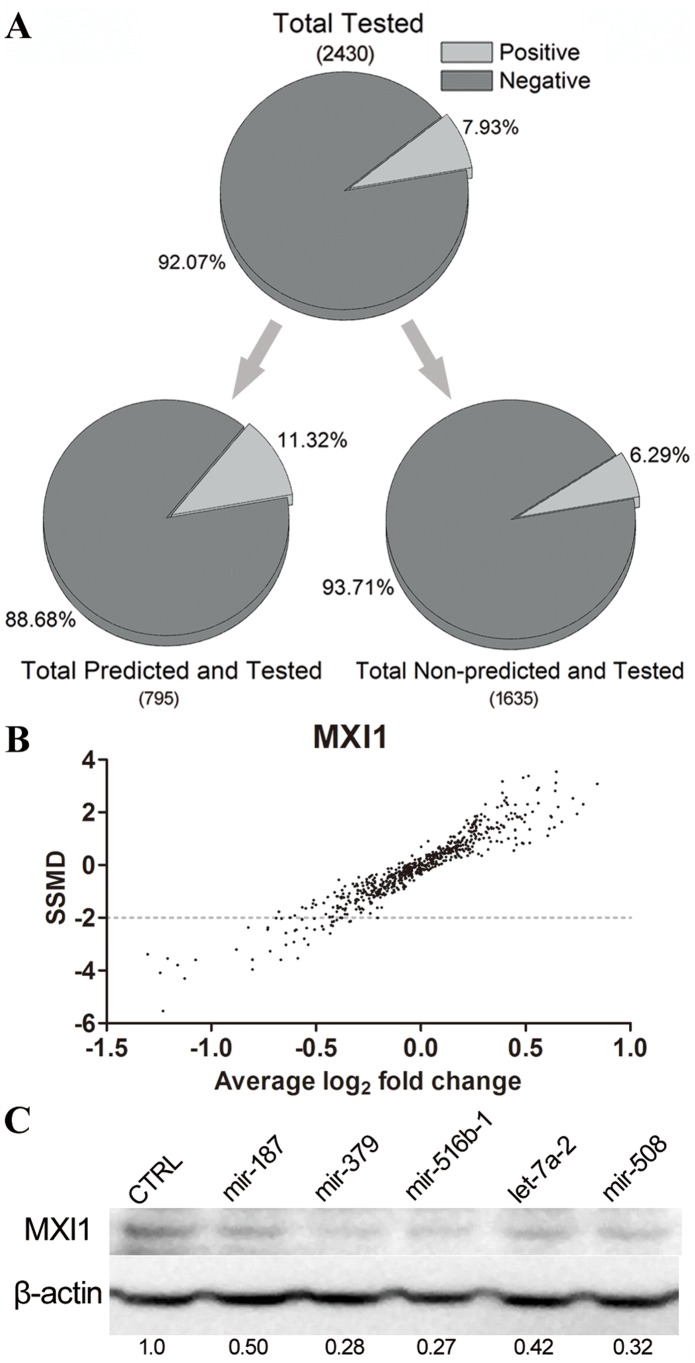
Large-scale screens of genome-wide miRNAs that may target the 3′UTRs of the four genes. (A) About 611 pre-miRNAs in the expression library were used to screen the miRNAs that could target each 3′UTR of the four genes *MXI1*, *POT1*, *TRF2* and *FSCN1*. The data for the four genes were combined to evaluate the percentages of positive in the total tested interactions, predicted interactions and unpredicted interactions. (B) SSMD-fold change dual-flashlight dots was used to analyze the screening data of MXI1. Fold changes were calculated according to the negative controls in each plate. (C) Immunoblotting was used to examine the endogenous protein level of *MXI* in 293T cells when the inhibitive miRNAs were transfected into the cells and cultured for 48 h post-transfection.

To test whether there were unpredicted miRNA-mRNA 3′UTR interactions, we performed genome-wide screens of *MXI1, POT1, TRF2* and *FSCN1* genes, with the constructed miRNA expression library. For each gene, every miRNA expression vector (about 600 in total), whether predicted to bind the gene 3′UTR or not, were co-transfected with the luciferase reporter containing the full-length 3′UTR of the gene (an empty miRNA expression vector or rno-miR-344a-3p expression vector expressing mature miRNA with no human homology was used as a negative control) in 293T cells. Among 2,430 tested interactions, 193 positive interactions were found with a significant difference (*P*<0.05), accounting for 7.9% of all tested interactions (**Figure 5A**, upper pie chart). Among 795 predicted interactions, 90 (11.3%) were positive (**Figure 5A**, lower left pie chart), while 103 (6.3%) of 1,638 unpredicted interactions were positive (**Figure 5A**, lower right pie chart). These results demonstrated that one gene’s 3′UTR can be targeted by multiple miRNAs. For MXI1, individual miRNAs were shown by SSMD-fold change dual-flashlight dots (**Figure 5B** **)**. Forty-two miRNAs were found to have SSMD values under −2, indicating a strong interaction [32]. Five unpredicted miRNAs, which down-regulated the *MXI1* 3′UTR in the screening results, were chosen for further confirmation by immunoblotting and all significantly decreased the level of endogenous *MXI1* protein (**Figure 5C**). Collectively, our results showed that there were many positive miRNA-mRNA interactions that were not predicted by 9 commonly used programs.

### Distribution and Conservation of miRNA Target Sites

**Figure 6:**
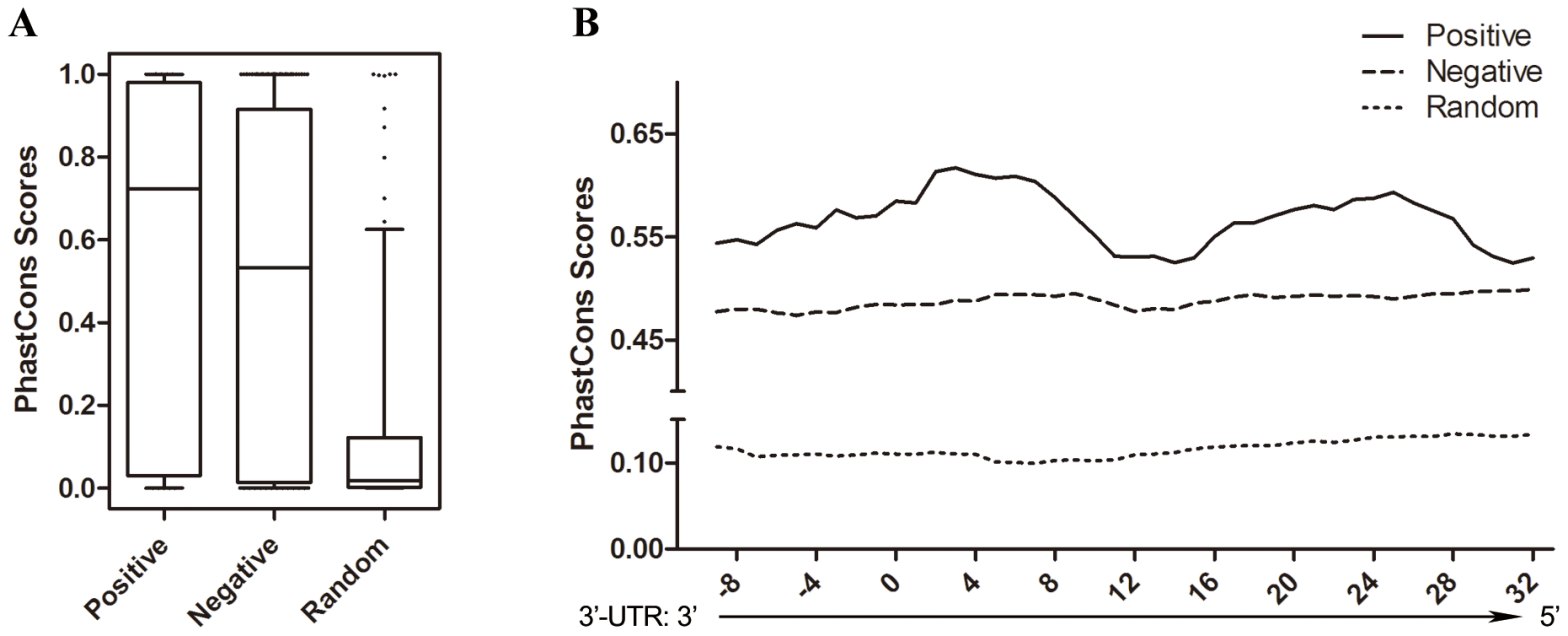
Conservation analysis of validated miRNA target sites. (A) The distribution of the average PhastCons score of predicted MREs in positive (n = 101), negative (n = 875) and random (n = 203) groups. There were significant differences between positive and negative groups as well as between positive and random groups (Wilcoxon test, p<0.05). (B) The PhastCons score of every nucleotide of MREs and boundary nucleotides in the three groups. The interactions with a SSMD value equal to or less than −3, which indicated the threshold of extremely strong inhibitive effects, were grouped to be positive. The nucleotides targeted by the miRNA seed region were designated as 2–8.

miRNA target sites were validated using conservation analysis to characterize the miRNA target elements (MREs). The predicted miRNA target sites were divided into positive group and negative group. Randomly chosen sequences of the human genome were used as control group. The average PhastCons score of positive group and negative group was 0.57 and 0.49 respectively, while that of the random group was only 0.12 (**Figure 6A**). The sequences of MREs in positive group were more conserved than those in the other two groups (Wilcoxon test, *P*<0.05). The conservation level of each single nucleotide was higher in positive group than in the other two groups. Two conserved regions contained in positive group that were not found in the negative or random group. One was miRNA seed region target sites, 2–8 nucleotides, and the other was in the 3′ end of miRNA target region, 14–25 nucleotides. However, the region between them was less conserved (**Figure 6B**).

## Discussion

In the present study, using the dual luciferase assay in large-scale screens, many miRNA-mRNA interactions were identified, including predicted and unpredicted interactions. Some genes were found to have been regulated by multiple miRNAs. The results of this study also demonstrate that, in addition to the complementary sequences of the miRNA seed region, a relatively high conservation region (i.e., the miRNA 3′ end targeted region) is also important to miRNA targets.

### Large-scale Methods for the Identification of miRNA Targets

We showed that EIMMo had the highest prediction precision (27%) and miRDB had secondly highest precision (24.7%), and the positive interaction ratio was 6.3% in the unpredicted group. Co-IP based methods are widely used to identify miRNA targets, which are often combined with miRNA transfection experiments [41]. Transcriptome-wide identification of RNA-binding proteins (RBPs) and miRNA target sites by photoactivatable-ribonucleoside-enhanced crosslinking and immunoprecipitation (PAR-CLIP) allows high-resolution mapping of binding sites of cellular RBPs and microRNA-containing ribonucleoprotein complexes (miRNPs) across the transcriptome [4], [42]. Co-IP based methods have some flaws although they are effective to identify miRNA targets. For example, in the sequence clusters, it is unable to identify each miRNA’s direct target. In addition, these co-IP based methods require a stable association between AGO, miRNA and targets. Some miRNA-mRNA interactions cannot be identified probably because a subset of miRNAs and/or targets do not survive during the washing steps of miRNP complexes [26]. We tested every miRNA with its target mRNA using only a full-length 3′UTR cloned in a luciferase reporter gene, which could partially rule out some indirect effects. However, we observed that several miRNAs led to increase luciferase activity. The cause of this is probably because microRNAs and their associated protein complexes (microribonucleoproteins or microRNPs) could function to posttranscriptionally stimulate gene expression by relief of repression where the action of a repressive microRNA or microRNP is abrogated. Although there are some issues just as we are not sure whether every vector could produce mature miRNA for the library, our approach could generally identify each miRNA’s target and avoid the interactions that were unpredicted using other methods.

### One Gene Targeted by Multiple miRNAs

MiRNA target prediction provides a clue for experimental identification of miRNA-mRNA interactions. Ideally, high-throughput experiments would give concise answers in simple over-expression experiments. Unfortunately, this is not entirely the case [43]. To identify the miRNA target genes, we performed a systematic experiment to understand miRNA-RNA interactions. We chose 4 genes for a large-scale screening experiment. For each gene, about 600 miRNA expression vectors were transfected individually with luciferase reporter into the cells. It has not been previously reported partially because of the large scope of the task. We found many miRNAs could target one gene’s mRNA. It has been reported that a single miRNA can target many genes, while a single gene can be regulated by multiple miRNAs [44], [45]. In a study of miRNAs targeting p21/Waf, 28 of 266 miRNAs were reported to target p21/Waf1. These miRNAs can substantially inhibit p21/Waf1 expression, particularly at the translational level. The results were then verified by a series of mutational analyses and luciferase assays [44]. In another study, 7 of 45 miRNAs were found to repressed a CyclinD1 3′’UTR luciferase reporter [46]. It has been suggested that, in particular tissues or developmental stages, these miRNAs might have both spatial and/or temporal specificities [44]. Moreover, the potential additive effect of 2 (or more) miRNAs targeting the same 3′UTR would be also interesting. For example, in the study of Hand2, the authors found that miR-1 and miR-133a can act together on the 3′UTR of Hand2 to produce addictive or synergistic effects on protein production [45]. To our knowledge, so far, it is the first systematic large-scale screening with the task of large scope.

### Evaluation of Prediction Software

Although many miRNA prediction programs have been developed, they are insufficient for use as efficient and powerful tools. An appropriate database is the essential tool for this purpose. Alexiou *et al*. [47] compared some prediction programs with analysis of a proteomic dataset obtained by Selbach *et al*. [27]. Although the dataset was based on the changes in protein levels with specific miRNA overexpression or endogenous miRNA knockdown, we know less about the direct relationship of the changes between protein levels and miRNA expression levels [48]. In this study, our dataset was obtained from the interactions between the miRNAs and the full-length 3′UTRs of coding genes. The only full-length 3′UTR cloned in the luciferase reporter gene is capable of ruling out some indirect effects, such as the change in protein levels that arise from endogenous miRNA-target transcriptional factors, which may regulate many target genes. Although every miRNA was tested with one gene’s 3′UTR, indirect effects could not be completely ruled out in our interaction validation system. The dataset from this system is already much closer to direct interaction, which was demonstrated by the MRE site mutation analysis and immunoblotting experiments.

All 9 miRNA prediction programs used in this study showed low prediction efficiencies. Among them, EIMMo and miRDB gave relatively higher precision than the others. EIMMo was designed based on a Bayesian phylogenetic miRNA target identification algorithm, which could predict miRNA target sites, and each site could be ranked by a posterior probability [22]. miRDB was designed based on a support vector machine (SVM) algorithm and the rules, such as seed conservation, seed match type, 3′UTR base composition, the secondary structure of the miRNA binding and the target site location in the 3′UTR are the classifier features [20], [21]. Although most of these features have been used by other prediction programs, miRDB appears to have a better performance than others. Our results indicated that these kinds of algorithms may be more suitable for exploitation of miRNA prediction.

### Pattern of MREs

Sequence conservation is a well-known character of miRNAs, especially in the seed region and the 13–16 nt positions of miRNA [4], [13]. We found that the MREs of the positive interactions were relatively more conservable than the MREs of the negative and random interactions (**Figure 6A**). Our results demonstrated two relatively conserved regions in the miRNA target sites. One corresponds to the seed region and the other is the 3′ end of the miRNA targeting segment. The conservation pattern is found only among the positive MREs, but not in the other two groups. The finding for the 3′ end of the miRNA targeted region is consistent with the results from *C. elegens*, in which the artificial 3′UTR containing only the miRNA seed site was totally out of control of the miRNA [49]. Some single mutations introduced in the positions of the miRNA 3′ end targeted 3′UTR can also impact regulation [50]. Several studies indicated that the RNA folding of both local segment and global 3′UTR could affect the prediction precision [51], [52], which implicated that the second structure of miRNA binding region as well as surrounding region could be contributed to the authentic miRNA target. This may be the reason that the higher conservation level in that region extends after the end of the miRNA binding sequence. Collectively, the features of miRNA targeted sequences may shed some light on the development of new prediction tools.

In summary, we have performed a large-scale screening method to identify miRNA targets and provided an option to evaluate the prediction programs. Meanwhile, we demonstrated that mRNA of one gene could be targeted by multiple miRNAs. This is an important improvement in the study of miRNAs in human disease for the future.

## Supporting Information

Table S1 **Primers used for amplifying 3′UTRs.**
(XLS)

Table S2 **Synthesized oligos.**
(XLS)

Table S3 **Prediction data for seven genes.**
(XLS)

Table S4 **Prediction software.**
(XLS)
